# Conditional validity in LLM-mediated L2 assessment: an argument-based systematic review and meta-analysis

**DOI:** 10.3389/frma.2026.1908592

**Published:** 2026-09-11

**Authors:** Latifah Hamdan Alghamdi, Talal Musaed Alghizzi

**Affiliations:** 1 ELC, King Khalid University, Abha, Saudi Arabia; 2 College of Languages and translation, Imam Mohammad ibn Saud Islamic University (IMSIU), Riyadh, Saudi Arabia

**Keywords:** AI-generated feedback, argument-based validity, automated scoring, fairness, human–AI assessment, L2 assessment, large language models, reliability

## Abstract

**Introduction:**

Large language models (LLMs) are increasingly used for scoring and feedback in second-language (L2) assessment, yet the validity of the resulting interpretations remains contested. This review evaluated when LLM-mediated assessment is psychometrically and educationally defensible using an argument-based validity framework focused on construct representation, reliability and reproducibility, fairness, and washback.

**Methods:**

Following PRISMA 2020, we systematically searched nine databases and repositories for empirical studies published from January 2022 to December 2025. Fifty-two studies met the inclusion criteria. Quantitative synthesis used REML random-effects models where effects were sufficiently comparable; Pearson correlations, rank correlations, ICC/κ/QWK, and other psychometric indices were otherwise retained on metric-appropriate scales. Washback outcomes were synthesized using Hedges' g.

**Results:**

Source-level auditing showed that directly comparable Pearson evidence was sparse. Three source-verified L2 studies yielded moderate human-LLM score correspondence (*r* = 0.66, 95% CI [0.53, 0.75]) with substantial heterogeneity (*I*^2^ = 70.8%). Evidence was strongest in rubric-guided, structurally constrained, and human-supervised contexts, whereas construct representation, fairness, operational reproducibility, and generalizability remained underdeveloped. Across 14 studies, LLM-mediated formative feedback produced a small-to-moderate positive effect on revision quality and short-term writing outcomes (*g* = 0.42, 95% CI [0.27, 0.57]), although heterogeneity was substantial (*I*^2^ = 69.8%) and concerns about cognitive offloading, learner agency, and reproducibility persisted.

**Discussion:**

The findings support a context-dependent interpretation of validity rather than a universal claim about LLM assessment capability. Current evidence supports carefully bounded formative and human-supervised applications but does not justify autonomous high-stakes scoring or broad claims of construct validity, fairness, and generalizability.

## 1 Introduction

Large language models (LLMs) are increasingly used in second language (L2) assessment to generate scores, provide feedback, classify proficiency, and support rubric-based evaluation (Emirtekin, 2025; Gjorevski et al., 2025; Huang and Wilson, 2025). Their expanding role represents a significant change in educational assessment practice: artificial intelligence (AI) is increasingly used not merely as an instructional aid but as a contributor to interpretations of learner ability and, in some contexts, to consequential assessment decisions (Wang et al., 2025). Consequently, the central question is not whether LLMs can approximate human ratings, but whether the inferences derived from their outputs are valid, reliable, fair, and educationally defensible.

This question poses challenges that differ from those associated with earlier automated scoring systems. Traditional automated assessment relied on predefined and auditable linguistic features within relatively stable scoring pipelines (Hackl, 2023). By contrast, LLMs generate probabilistic outputs that may vary across prompts, interaction histories, model versions, and deployment conditions (Kim, 2025; Song et al., 2025; Wang et al., 2025). Such variability raises concerns about construct representation, reproducibility, transparency, and score comparability that agreement statistics alone cannot resolve (Gaggioli et al., 2025; Huang and Wilson, 2025). Although existing research reports encouraging results for formative feedback, rubric-guided scoring, and revision support (Asadi et al., 2025; Ding, 2025; Guo and Wang, 2024; Han and Li, 2024), the evidence remains concentrated on human–AI agreement and short-term performance outcomes (Yang, 2024). Far less attention has been devoted to whether LLM outputs consistently represent intended constructs, function equitably across learner populations, or produce appropriate educational consequences (Blodgett et al., 2024; Gallegos et al., 2024). This distinction is critical because agreement with human raters, while necessary, is not sufficient evidence of validity.

To address these gaps, this systematic review and meta-analysis synthesize empirical research published between January 2022 and December 2025 through an argument-based validity framework (Kane, 2013). The review evaluates evidence across four inferential domains: construct representation, reliability and reproducibility, fairness and subgroup comparability, and washback and consequential validity. Adopting a conditional validity perspective, the argument is that validity in LLM-mediated assessment is not an inherent property of the technology itself but depends on the assessment purpose, construct complexity, operational conditions, prompting architecture, and human oversight. Current evidence supports carefully bounded use in formative, rubric-guided, and human-supervised contexts. However, it remains insufficient to justify autonomous high-stakes deployment or to support universal claims of validity and fairness across diverse settings.

### 1.1 Rationale and significance

The rapid adoption of LLMs in L2 assessment reflects growing demand for scalable scoring and feedback systems, particularly for writing and speaking tasks that require substantial human expertise and resources (Emirtekin, 2025). However, assessment defensibility depends on more than efficiency or score agreement. Defensible assessment requires evidence that scores represent intended constructs, remain reproducible across conditions, function equitably across learner populations, and support appropriate educational consequences (Kane, 2013; Messick, 1989).

Current evidence remains uneven. Support is strongest in structured, rubric-guided, low-stakes, and human-supervised contexts. In contrast, evidence is weaker for higher-order communicative judgment, fairness across diverse populations, and long-term educational consequences. This review addresses that imbalance by synthesizing evidence on validity, reproducibility, fairness, and washback within a unified, argument-based framework. Its contribution is not to determine whether LLM-mediated assessment is universally valid, but to clarify the conditions under which validity claims are supported and where evidential limitations remain.

### 1.2 Objectives and research questions

This review synthesizes empirical studies published between January 2022 and December 2025 on the use of LLMs in L2 assessment and feedback. Moving beyond score agreement and learner satisfaction as standalone indicators of quality, the review evaluates whether current evidence supports valid, reliable, fair, and educationally defensible interpretations of LLM-mediated assessment results. It is organized around the following research questions:

RQ1: What forms of validity evidence have been reported for LLM use in L2 assessment and feedback, and to what extent do they support construct representation beyond surface scoring correspondence?

RQ2: To what extent are LLM-generated scores and feedback reliable, stable, and reproducible across prompts, tasks, learner populations, model configurations, and deployment conditions?

RQ3: What fairness and bias issues have been examined in LLM-mediated L2 assessment and feedback, and what types of differential functioning remain unexamined?

RQ4: What washback and consequential effects are associated with LLM-generated feedback, particularly regarding revision quality, learner agency, metacognitive engagement, and authorship?

Together, these questions position validity as a structured evidentiary argument rather than a single statistical outcome. The review explicitly distinguishes score agreement, which supports limited scoring inferences from construct representation, and treats fairness, reproducibility, and washback as core components of assessment defensibility rather than secondary considerations.

## 2 Conceptual and theoretical framework

### 2.1 Argument-based validity in LLM-mediated assessment

This review is grounded in argument-based validity theory (Kane, 2013; Messick, 1989), which conceptualizes validity as the defensibility of score interpretations and uses rather than a property of a test or technology. Validity depends on a chain of interpretive inferences linking observed performance to claims about learner ability and educational decision-making. Weakness in any inferential link limits the overall validity of the argument. Applied to LLM-mediated assessment, this perspective shifts attention from whether LLMs approximate human ratings to whether their outputs support defensible interpretations under intended conditions of use. Human–LLM agreement informs the scoring inference but does not, by itself, establish construct representation, generalizability, fairness, or appropriate educational consequences. Agreement is therefore treated as necessary but insufficient evidence of validity.

Unlike earlier automated scoring systems, LLMs generate probabilistic outputs that may vary across prompts, model versions, and deployment conditions (Vaswani et al., 2017). These characteristics expand assessment capabilities while introducing additional evidential demands regarding construct representation, reproducibility, and subgroup comparability. This evidential burden connects argument-based validity theory to a broader, and more recent, literature on the accountability and governance of algorithmic systems, which similarly treats predictive and generative models as sociotechnical systems whose outputs cannot be evaluated on statistical performance alone but must be assessed against the institutional context, population, and consequences of their deployment (Blodgett et al., 2024; Gallegos et al., 2024).

The present framework draws on this literature to motivate why subgroup comparability and deployment-condition sensitivity, not only human–model agreement, belong in the validity argument for LLM-mediated assessment; it does not treat that literature as a substitute for argument-based validity theory itself, which remains the review's primary theoretical foundation. It is further informed by emerging scholarship on algorithmic accountability (Raji et al., 2020), explainable AI (Barredo Arrieta et al., 2020), sociotechnical AI governance (Selbst et al., 2019), algorithmic fairness (Blodgett et al., 2024; Gallegos et al., 2024), dynamic evaluation of foundation models (Chang et al., 2024), and foundation-model assessment (Bommasani et al., 2021). Collectively, this literature emphasizes that AI systems should be evaluated not only for predictive performance but also for transparency, robustness, interpretability, fairness, and context-sensitive deployment. These perspectives complement argument-based validity theory by reinforcing that validity claims for LLM-mediated assessment depend jointly on psychometric evidence and the documented behavior of the underlying AI system under its intended conditions of use.

### 2.2 Construct representation and the limits of agreement

Construct representation concerns whether LLM evaluations reflect the language abilities they are intended to assess rather than proxy features that correlate imperfectly with those abilities. Existing evidence suggests stronger alignment with expert raters on lower-inference dimensions such as grammatical accuracy and vocabulary use than on discourse-level, rhetorical, and pragmatic judgments (Hackl, 2023; Tang et al., 2024; Huang and Wilson, 2025). Critically, agreement and validity are not synonymous. LLMs may reproduce human score patterns while relying on different textual cues, including lexical sophistication, text length, or structural regularity. Consequently, construct validity requires evidence that evaluations consistently reflect intended language constructs across tasks, learner populations, and operational conditions rather than merely demonstrating score correspondence.

### 2.3 Reliability, stability, and reproducibility

Within LLM-mediated assessment, reliability extends beyond agreement with human raters to include consistency across prompts, repeated runs, model versions, and deployment settings (Kim, 2025; Song et al., 2025; Wang et al., 2025). Because outputs are probabilistic and context-sensitive, strong performance under controlled conditions does not necessarily generalize to operational use. Within Kane's (2013) interpretive argument, this concern maps most directly onto the generalization inference, which warrants moving from an observed performance to an expected score over the relevant universe of prompts, occasions, and raters, and onto the extrapolation inference, which warrants moving from that expected score to performance under operational conditions beyond the observed sample. What distinguishes LLM-mediated assessment is not that these inferences are new, but that the “universe of generalization” itself becomes unstable: prompt phrasing, model version, and decoding settings can shift the very population of outputs over which generalization is being claimed, in a way that has no direct analog in fixed-form human or earlier automated scoring. Reproducibility is therefore treated here as a validity-relevant requirement, a precondition for the generalization and extrapolation inferences to hold, rather than as a fifth, freestanding validity domain or a purely technical consideration. Reproducibility problems are more visible in LLM-mediated systems because of their probabilistic outputs. However, the underlying inferential requirement is the same one Kane's model already specifies for any measurement procedure.

### 2.4 Fairness as a validity concern

Fairness is an integral component of validity rather than a separate ethical issue (American Educational Research Association [AERA], American Educational Research Association, 2014; Messick, 1989). In LLM-mediated assessment, fairness concerns arise when comparable ability can yield different outcomes across learner groups due to differential scoring behavior, uneven training-data representation, or culturally specific evaluation norms. This review organizes fairness evidence into three categories: measurement bias, representational bias, and construct-relevant cultural variation. Distinguishing among these forms of bias clarifies the evidential requirements of fairness claims and provides a framework for evaluating subgroup comparability in LLM-mediated L2 assessment.

### 2.5 Washback and consequential validity

Consequential validity concerns how assessment influences learning, revision practices, and engagement with feedback (Alderson and Wall, 1993; Messick, 1989). In LLM-mediated assessment, feedback may support more timely, individualized, and scalable revision, particularly in formative contexts (Asadi et al., 2025; Ding, 2025). However, feedback effectiveness should not be equated with positive washback. Improvements in revision quality or short-term performance do not necessarily indicate that the assessment supports desirable learning processes or long-term language development.

Of particular relevance are concerns regarding cognitive offloading, reduced metacognitive engagement, passive acceptance of automated revisions, and diminished authorial agency (Liu et al., 2024; Teng, 2024). Consequently, consequential validity requires attention not only to performance outcomes but also to the extent to which LLM-mediated feedback supports active, reflective, and agentive engagement with language learning.

### 2.6 Integrative framework and conditional validity

**Figure 1:**
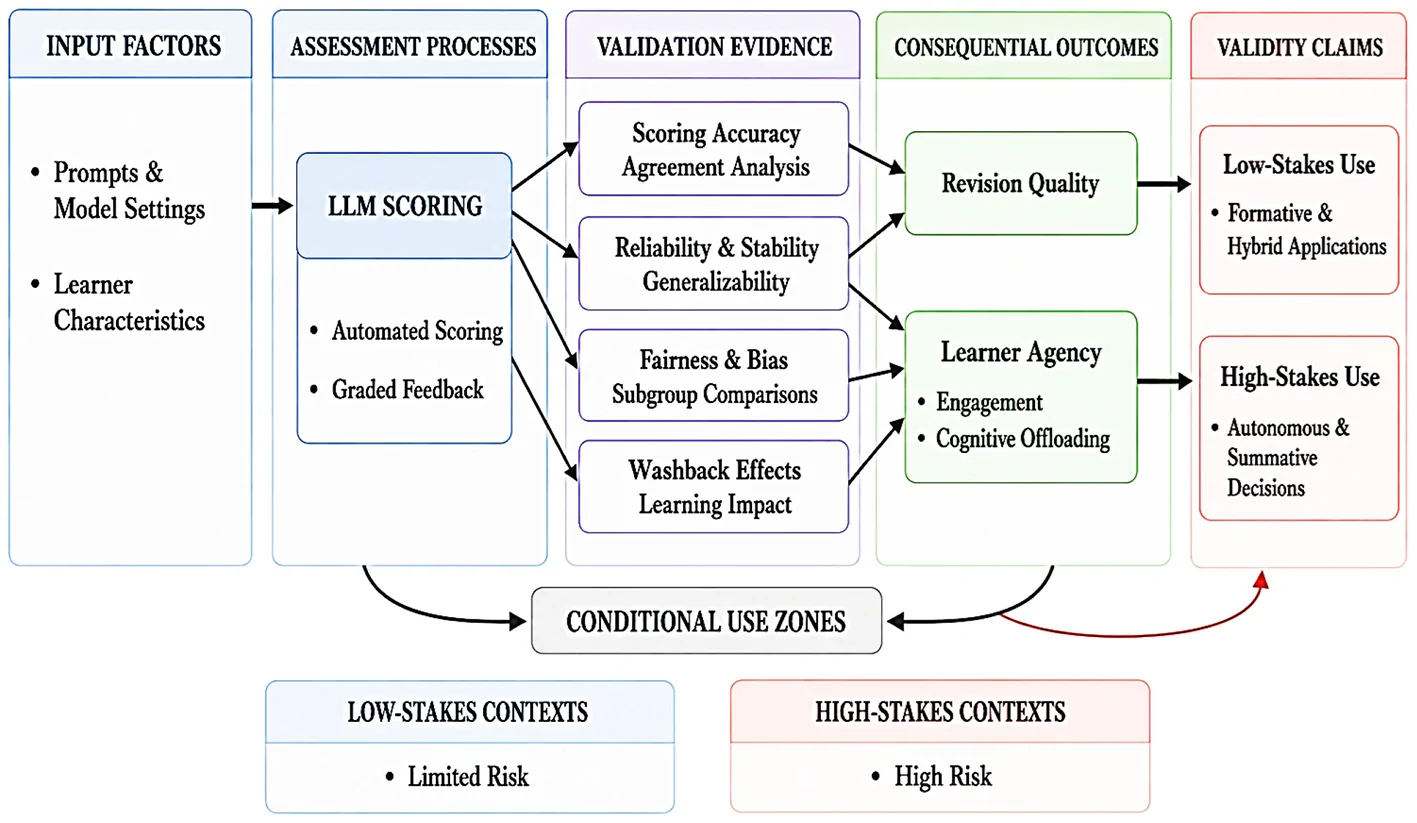
Argument-based validity framework for LLM-Mediated L2 assessment.

This review treats construct representation, reliability and reproducibility, fairness, and washback as interconnected components of a unified validity argument. Evidence in one domain cannot compensate for weaknesses in another. High agreement may coexist with limited construct representation, positive revision outcomes may occur alongside reduced learner agency, and apparent fairness may reflect insufficiently sensitive subgroup analyses. Accordingly, the review adopts a conditional validity perspective (Figure 1), using conditional as a concise label for the established principle that validity is contingent on context, population, intended use, and interpretive assumptions within argument-based validity theory (Messick, 1989; Kane, 2013; AERA, APA, and NCME, 2014). The term does not denote a distinct validity construct or alternative validity theory. Rather, it describes the context-dependent strength of validity arguments in LLM-mediated assessment under varying deployment conditions. Contemporary validity scholarship (Messick, 1989; Kane, 2013; AERA, APA, and NCME, 2014) already conceptualizes validity as contingent on context, population, and intended use; the term *conditional* is therefore used as shorthand for this context dependence.

Figure 1 should be interpreted as a decision-support heuristic that operationalizes, rather than extends, Kane's interpretive argument by organizing the evidence required to support validity claims in LLM-mediated assessment. It introduces no new validity warrants; instead, construct underrepresentation, inadequate reproducibility, insufficient fairness evidence, and adverse consequential effects function as rebuttal conditions that weaken the validity argument despite high human–LLM agreement. Accordingly, validity claims are proportional to evidential convergence: carefully bounded formative applications may be defensible when evidence converges across construct representation, reproducibility, fairness, and washback under specified conditions of use, whereas autonomous high-stakes deployment requires substantially stronger evidence across all four domains. The framework's contribution is therefore organizational and domain-specific rather than paradigmatic, providing a principled basis for judging the strength and appropriate scope of validity claims in AI-mediated L2 assessment rather than introducing a new inferential architecture.

## 3 Review protocol and transparency

### 3.1 Review design and protocol development

This study followed a predefined protocol developed before searching, screening, and data extraction. The review adhered to the Preferred Reporting Items for Systematic Reviews and Meta-Analyses (PRISMA) 2020 guidelines (Page et al., 2021) and was organized within the argument-based validity framework outlined in Sections 2.1–2.6. Eligibility criteria, search procedures, extraction protocols, and planned quantitative synthesis strategies were specified a priori. Methodological refinements and the post-review quantitative audit are documented in **Appendix C**. The audit preserved the review questions and theoretical framework while revising how heterogeneous score-comparison statistics were synthesized, retaining only source-verifiable and metric-appropriate analyses.

### 3.2 Transparency and reproducibility procedures

Screening, extraction, and analysis procedures were standardized to enhance transparency and reproducibility. Dual-reviewer screening yielded strong inter-rater agreement (κ = 0.84), with disagreements resolved through discussion and, when necessary, third-reviewer adjudication. Data extraction used standardized coding matrices, and a 20% subsample was independently double-coded to verify consistency. Because reproducibility is central to the review's validity framework, extraction procedures explicitly documented prompt transparency, model-version reporting, parameter settings, and repeated-run procedures. Incomplete reporting of these variables was common across the literature and is treated as a substantive finding relevant to interpreting current evidence. Table 1 summarizes the review architecture, transparency procedures, and synthesis strategy.

**Table 1:** Methodological architecture of the review.

Review component	Procedures implemented
Protocol and reporting	PRISMA 2020-guided; predefined protocol; deviations documented in **Appendix C**.
Screening procedures	Dual-reviewer independent screening; κ = 0.84; adjudication procedure for unresolved disagreements.
Data extraction	Standardized coding matrices; 20% double-coded subsample; reproducibility variables coded explicitly.
Reproducibility procedures	Prompt documentation, model-version tracking, parameter coding, repeated-run recording.
Quantitative synthesis	REML random-effects for source-verified direct Pearson correspondence and washback; Pearson + Spearman sensitivity analysis; native ICC/κ/QWK and other psychometric metrics retained separately.
Heterogeneity assessment	Cochran's Q, *I*^2^, and τ^2^ for pooled outcomes; prediction intervals only where *k* and model structure make them interpretable.
Publication bias	Egger/trim-and-fill only for independent syntheses with adequate *k*; retained for washback (*k* = 14), not for direct correspondence (*k* = 3).
Transparency resources	Search strings (**Appendix A**); PRISMA checklist (**Appendix B**); protocol deviations (**Appendix C**).

## 4 Methods

### 4.1 Review design and analytical orientation

Building on the protocol established in Section 3, this section specifies the study designs, eligibility criteria, search strategy, extraction procedures, and meta-analytic methods used to synthesize empirical research on LLM-mediated L2 assessment and feedback. The review integrated quantitative meta-analysis with structured qualitative synthesis to accommodate methodological diversity across the evidence base. Guided by the argument-based validity framework outlined in Sections 2.1–2.6, the review evaluated evidence across four domains: construct representation, reliability and reproducibility, fairness, and washback. Table 2 summarizes the review's analytical structure.

**Table 2:** Analytical structure of the review.

Inferential domain	Primary analytical focus
Construct representation	Alignment between LLM outputs and intended language constructs across tasks, populations, and conditions
Reliability and reproducibility	Prompt consistency, repeated-run stability, model-version comparability, and inter-rater agreement
Fairness and subgroup comparability	Differential scoring severity, feedback equity, demographic sensitivity, and bias type classification
Washback and consequential validity	Revision quality, learner agency, metacognitive engagement, feedback uptake, dependency risks

### 4.2 Eligibility criteria

Eligible studies were empirical investigations of LLM-mediated assessment or feedback involving L2, English as a foreign language (EFL), English as a second language (ESL), multilingual, heritage-language, or additional-language learners across secondary, higher education, adult, and professional contexts. The review covered studies published between January 2022 and December 2025, reflecting the emergence and rapid adoption of generative LLMs in educational assessment.

Eligible designs included experimental, quasi-experimental, correlational, validation, mixed-methods, and qualitative empirical studies. Peer-reviewed articles, dissertations, conference proceedings, and publicly accessible preprints were retained if they reported analyzable evidence on construct representation, reliability and reproducibility, fairness, or washback. Publication status was coded for all studies, and sensitivity analyses examined whether findings differed between peer-reviewed and non-peer-reviewed sources. Table 3 summarizes the eligibility framework.

**Table 3:** Eligibility framework.

Component	Inclusion criteria
Population	L2, EFL, ESL, multilingual, heritage-language learners across educational levels
Assessment function	Automated scoring, formative feedback, revision mediation, diagnostic assessment, human–AI collaborative rating
Outcomes	Validity, reliability, fairness, reproducibility, or washback evidence—quantitative, qualitative, or mixed
Study designs	Experimental, quasi-experimental, correlational, validation, mixed-methods, qualitative empirical
Publication types	Journal articles, dissertations, conference proceedings, peer-reviewed preprints
Publication period	January 2022–December 2025

### 4.3 Exclusion criteria

Studies were excluded if they lacked primary empirical data, focused exclusively on instructional AI rather than assessment functions, did not involve L2 learners or contexts, or provided insufficient methodological information for extraction and interpretation. Conceptual papers, editorials, and technical system descriptions were excluded unless they reported analyzable empirical evidence. Studies focusing solely on L1 assessment were excluded. Duplicate reports based on the same dataset were consolidated, retaining the most complete or methodologically transparent source.

### 4.4 Search strategy and study selection

A comprehensive search was conducted across nine databases and repositories: Web of Science, Scopus, ERIC, APA PsycINFO, LLBA, ProQuest Dissertations and Theses Global, IEEE Xplore, ACM Digital Library, and arXiv. Search strings combined LLM-related terms (e.g., ChatGPT, GPT-4, large language model, generative AI), assessment-related terms (e.g., scoring, feedback, validity, reliability, fairness, washback), and L2-related terms (e.g., second language, EFL, ESL, L2 writing, L2 speaking). Complete search strategies are provided in **Appendix A**.

**Figure 2:**
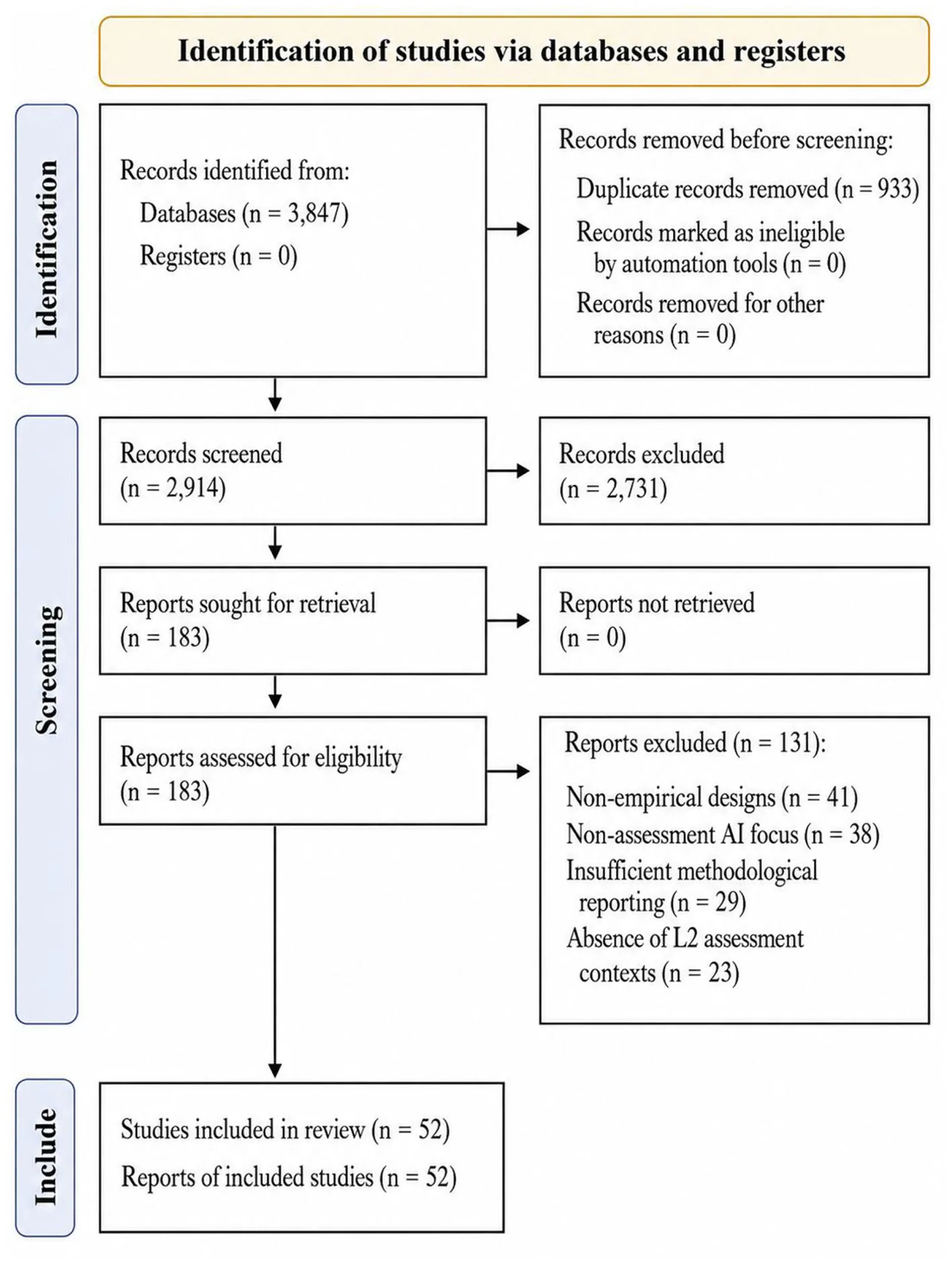
PRISMA flow diagram of the study selection process.

Searches were conducted in December 2025. Forward and backward citation chaining was performed on all included studies. Study selection followed a PRISMA 2020-compliant process involving deduplication, title–abstract screening, full-text review, and final inclusion. Two reviewers conducted screening independently using piloted criteria, yielding strong inter-rater agreement (κ = 0.84). Figure 2 presents the study selection flow. The search yielded 3,847 records, of which 2,914 remained after deduplication. Following title–abstract screening, 183 records were sought for retrieval and assessed for eligibility in full text; seven were identified through forward and backward citation chaining conducted throughout the review process, and 176 through the nine-database search. Of these 183 records, 131 were excluded, primarily because of non-empirical designs (*n* = 41), non-assessment AI focus (*n* = 38), insufficient methodological reporting (*n* = 29), or absence of L2 assessment contexts (*n* = 23), yielding a final corpus of 52 empirical studies.

### 4.5 Data extraction and quality appraisal

Data extraction employed standardized coding matrices piloted before formal extraction. Extracted variables included study characteristics, participant profiles, assessment functions, LLM configurations, validity-related outcomes, fairness indicators, and washback findings. Reproducibility-relevant variables, prompt transparency, model-version reporting, parameter settings, and repeated-run procedures, were coded using a four-level transparency rubric (full, partial, minimal, or absent documentation). Methodological quality was evaluated using the Mixed Methods Appraisal Tool (MMAT; Hong et al., 2018). Appraisal considered sampling adequacy, reporting transparency, statistical appropriateness, subgroup rigor, and reproducibility documentation. Quality ratings informed sensitivity analyses but were not used as exclusion criteria due to the methodological diversity and the emerging nature of the evidence base.

### 4.6 Meta-analytic procedures

Quantitative synthesis employed random-effects models estimated via restricted maximum likelihood (REML; Viechtbauer, 2010). Following the post-review source-level audit, the previously reported cross-metric 21-study score-correspondence composite was not retained because the exact historical study-level inputs and effect-specific ICC/κ-to-r conversion procedure could not be independently reconstructed from the archived materials. The primary reproducibility re-analysis was therefore restricted to source-verified Pearson correlations from strictly eligible L2 studies for which the reported coefficient, analytic sample size, and source locator were recoverable. Pearson correlations were Fisher-z transformed, with sampling variance based on 1/(n-3), and back-transformed for interpretation. A separate sensitivity analysis added two source-verified Spearman rank correlations without relabeling them as Pearson correlations. ICC, weighted κ/QWK, generalizability-theory (G-theory) coefficients, and many-facet Rasch (MFRM) indices were retained on their native scales and synthesized descriptively or metric-specifically because they represent different measurement properties. Washback outcomes continued to be synthesized using Hedges' g. The complete source-level audit and effect calculations are provided in Supplementary Table 8.

For the direct Pearson re-analysis, one study-level coefficient was used per source in the conservative reproducibility check so that multiple model-, occasion-, or rubric-specific coefficients from the same participants were not treated as independent studies. Heterogeneity was summarized using Cochran's Q, *I*^2^, and τ^2^. Prediction intervals were reported only when the number of independent studies and model structure made them interpretable; for the k = 3 direct-Pearson re-analysis, the t-based prediction interval was effectively uninformative and is therefore not emphasized; see Table 4. A same small-k constraint that limits the prediction interval also limits the precision of the REML τ^2^ and *I*^2^ estimates at *k* = 3; these are retained because the confidence interval around the pooled point estimate remains informative for describing the currently auditable evidence, but, consistent with the prediction-interval decision above, none of these heterogeneity statistics should be read as a stable characterization of population-level variability, and the *k* = 3 estimate is reported as a bounded, source-verified summary rather than a generalizable index of LLM scoring performance. Pearson's r is treated as a measure of linear association rather than exact-value agreement; ICC and κ/QWK are reported separately as agreement/reliability evidence.

**Table 4:** Meta-analytic framework.

Component	Procedures
Model type	Random-effects (REML estimation); multilevel where dependencies are present
Main outcomes	Direct score correspondence (Pearson r); rank-correlation sensitivity (Spearman ρ); native agreement (ICC/κ/QWK); reliability/reproducibility; fairness; washback (*g*).
Source and synthesis metrics	Primary auditable correspondence re-analysis: source-verified Pearson r values from strict L2 studies, Fisher-z transformed. Spearman ρ retained as rank correlation in sensitivity analysis only. ICC and weighted κ/QWK retained on native scales; MFRM and G-theory kept metric-specific. Washback: Hedges' *g*.
Dependency handling	One study-level Pearson coefficient per source in the conservative reproducibility recheck; multiple within-study effects not counted as independent studies. Multilevel/robust variance estimation (RVE) retained only where dependent effects are explicitly modeled.
Heterogeneity metrics	Cochran's *Q*; *I*^2^; τ^2^; prediction intervals only when k is sufficient and the interval is substantively interpretable.
Primary interpretive metric	Pooled estimate and 95% confidence interval (CI); heterogeneity; prediction interval only where estimable; metric-specific narrative evidence otherwise.

The post-review source-level audit replaced the unreproducible historical cross-metric composite with a source-verified direct-Pearson re-analysis. Spearman correlations are used only in a sensitivity analysis, while ICC and weighted κ/QWK remain on their native scales. No ICC or κ/QWK value is relabeled as Pearson's *r*. The asterisk on the native agreement row (*k* = 3^*^) denotes three source-verified studies reporting ICC or κ/QWK values; this is a distinct set of coefficients from the three studies pooled in the direct-Pearson row above, although one study (Liu et al., 2025) contributes an eligible statistic to both rows. Supplementary Table 8 provides the source statistic, value, *n*, locator, transformation, and analysis status for every audited effect.

### 4.7 Sensitivity, subgroup, and publication bias analyses

The source-verified direct-Pearson correspondence re-analysis contained only three independent studies, which was insufficient for defensible moderator meta-regression, publication-status comparisons, or leave-one-out influence claims of the type reported in an earlier version. Therefore, removed those correspondence-specific moderator results. A prespecified audit sensitivity analysis added two source-verified Spearman correlations to examine whether inclusion of rank-based association evidence materially changed the central estimate; these coefficients were kept explicitly labeled as Spearman ρ. Grammar-focused and discourse-level evidence was audited at the study/effect level but was not pooled because compatible, source-verifiable subgroup sets sufficient to reproduce the previously reported *k* = 11 and *k* = 9 syntheses could not be established.

Formal publication-bias tests were not conducted for the direct-Pearson correspondence re-analysis (*k* = 3) or the mixed Pearson-plus-Spearman sensitivity analysis (*k* = 5), because these sets were too small and, in the latter case, intentionally metric-heterogeneous. Publication-bias diagnostics were retained for the washback meta-analysis (*k* = 14), for which Egger's regression and trim-and-fill were reported. This revision avoids applying small-study-effect tests to a correspondence evidence base too small to support stable inference.

### 4.8 Certainty of evidence

Certainty of evidence was evaluated across the four inferential domains using four considerations: methodological rigor, consistency of findings, adequacy of subgroup representation, and transparency of reproducibility. Because conventional GRADE procedures were developed primarily for intervention research, a domain-specific framework was adopted.

The review considered GRADE-CERQual (Lewin et al., 2018), which adapts GRADE logic to qualitative evidence syntheses using four components: methodological limitations, coherence, adequacy of data, and relevance, as a closer methodological relative than intervention-focused GRADE. This review did not adopt it directly because CERQual was designed for syntheses of qualitative research findings, whereas this review combines a small source-verified correlation re-analysis, a quantitatively pooled washback outcome, and narratively or metric-specifically synthesized measurement evidence (agreement, fairness, construct representation, and reproducibility) within a single argument-based validity structure, and its core components therefore do not align directly with the evidential structure required here. The domain-specific framework used in this review instead borrows CERQual's logic of using multiple, explicitly defined considerations rather than a single quantitative algorithm, adapted to the methodological rigor, consistency, subgroup adequacy, and reproducibility-transparency considerations relevant to LLM-mediated assessment evidence specifically.

Evidence was classified as comparatively stronger, moderate, or limited. Comparatively stronger evidence reflected consistent findings across multiple methodologically adequate studies with sufficient transparency. Moderate evidence indicated generally consistent findings accompanied by notable methodological limitations, heterogeneity, or incomplete reporting. Limited evidence reflected sparse, inconsistent, or methodologically underpowered findings that restricted confident interpretation.

## 5 Results

### 5.1 Study selection and corpus characteristics

Following PRISMA 2020-guided searching, deduplication, and multi-stage screening, 52 empirical studies met all eligibility criteria and were retained for synthesis. The database search yielded 3,847 records, of which 2,914 remained after deduplication. Following title–abstract screening, 183 records were sought for retrieval and assessed for eligibility in full text, of which seven were identified through forward and backward citation chaining and 176 through the nine-database search. Of these 183 records, 131 were excluded because of non-empirical designs (*n* = 41), non-assessment AI focus (*n* = 38), insufficient methodological reporting (*n* = 29), or absence of L2 assessment contexts (*n* = 23), yielding a final corpus of 52 empirical studies (Table 5).

**Table 5:** Overview of the evidence base.

Component	Summary
Final included studies	52 empirical studies
Initial search yield	3,847 records across 9 databases
Post-duplication records	2,914 records
Full-text reviewed	183 records
Excluded from full-text	131 records (reasons documented)
Added via citation chaining	7 of the 183 full-text-reviewed records (not additional to them)
Publication period	January 2022–December 2025
Peer-reviewed	39 studies (75%)
Preprints	13 studies (25%)
Dominant context	University-level EFL writing assessment
Common designs	Experimental, correlational, mixed-methods, validation
Main assessment functions	Automated scoring, formative feedback, revision support
Smaller subdomains	Speaking assessment, integrated tasks, hybrid human–AI rating

Most studies examined university-level EFL writing assessment contexts. The evidence base was geographically distributed across East Asia, the Middle East, Europe, and North America, with fewer studies involving multilingual or mixed-L1 populations. Most studies were published in 2023 or 2024. Of the 52 included studies, 39 were peer-reviewed publications, and 13 were preprints. Publication status remained part of the extraction framework, but no publication-status meta-regression was retained for the source-verified direct-Pearson correspondence re-analysis because only three independent studies contributed to that estimate. In addition to the studies discussed individually elsewhere in this review, the included corpus also comprises Alanazi et al. (2025), Jaganov et al. (2025), Jin (2025), Jovic et al. (2025), Kashiha (2025), Kortemeyer (2024), Li H. et al. (2025), Li J. et al. (2025), Liew and Tan (2025), Ma et al. (2025), Mugaanyi et al. (2024), Polakova and Ivenz (2024), Schaller et al. (2024), Shan et al. (2025), Shermis (2025), Shi et al. (2026), Shin and Lee (2024), Stasuik (2025), Uto and Aramaki (2024), and Xiao et al. (2025), which contributed to the aggregate corpus counts, extraction tables, and quantitative synthesis reported throughout Sections 5.1–5.6 (Tables 5–9) without being individually discussed by name elsewhere in the narrative.

### 5.2 Assessment functions and reporting transparency

LLMs served multiple assessment-related functions across the 52 included studies: automated holistic and analytic scoring (*n* = 24), formative feedback generation (*n* = 18), revision mediation and iterative feedback (*n* = 14), diagnostic proficiency assessment (*n* = 7), and collaborative human–AI rating (*n* = 6). Several studies examined more than one assessment function, most commonly scoring and feedback. Reporting transparency varied across the corpus. Using the four-level transparency rubric, 18 studies (35%) fully documented prompts, model versions, and parameter settings; 19 (37%) provided partial documentation; 11 (21%) provided minimal documentation; and 4 (8%) provided insufficient information for reproducibility evaluation (percentages rounded). The most common reporting omission was prompt text, which was absent or only partially reported in 31 studies (60%). Model-version information was absent in 14 studies (27%), which referred only to “ChatGPT” or “GPT-4” without specifying version or access date.

Transparency classifications were incorporated into subsequent sensitivity analyses and reproducibility evaluations.

### 5.3 Distribution and quality of evidence

Evidence was unevenly distributed across the four inferential domains (Table 6). Construct representation and fairness were examined less frequently than score comparison and washback outcomes. Most studies operationalized validity primarily through comparisons with human ratings, but they reported those comparisons using heterogeneous metrics, including Pearson r, Spearman ρ, ICC, κ/QWK, generalizability-theory coefficients, and many-facet Rasch parameters; therefore, they were not treated as interchangeable. Methodological quality varied across the corpus. Common limitations included small sample sizes (median *N* = 67, range = 12–412), single-run designs, incomplete prompt reporting, limited subgroup analyses, and insufficient documentation of model versions and parameter settings. The source audit distinguished directly reproducible quantitative inputs from metric-specific or narratively synthesized evidence.

**Table 6:** Distribution of evidence across inferential domains.

Inferential domain	Studies	Quantitative status	Certainty level
Human–LLM score correspondence	42	3 direct-Pearson studies pooled; 2 additional rank-correlation studies in sensitivity; native ICC/κ/QWK separate	Limited for pooled direct-correlation estimate
Construct representation	11	Heterogeneous metric-specific evidence; no pooled construct meta-analysis retained	Limited
Reliability and reproducibility	18	5 repeated-run studies; narrative synthesis because metrics were incompatible	Limited
Fairness and subgroup comparability	9	6 quantitative subgroup studies reported; not pooled into a common effect	Limited
Washback and consequential validity	22	14 pooled using Hedges' *g*	Moderate (formative contexts)

### 5.4 Source-verified quantitative findings: human–LLM score correspondence

The post-review source-level audit showed that the historical 21-study cross-metric composite could not be independently reconstructed from the available study-level extraction materials, so the previously reported *r* = 0.74 estimate was removed. The study conducted a conservative random-effects re-analysis using three strictly L2, source-verified studies reporting directly usable Pearson correlations with recoverable sample sizes and locators: Pack et al. (2024), Yang (2024), and Liu et al. (2025). The exploratory pooled score-correspondence estimate was *r* = 0.66 (95% CI [0.53, 0.75]; τ^2^ = 0.022; I^2^ = 70.8%). With only three independent studies, the *t*-based prediction interval spanned almost the full possible correlation range and was not considered substantively interpretable; accordingly, no meaningful prediction interval is emphasized for this estimate. The result should therefore be read as a reproducibility-oriented summary of the currently auditable direct-Pearson evidence, not as a stable general estimate of LLM scoring performance.

A sensitivity analysis added two source-verified Spearman correlations (Mizumoto et al., 2024; Uyar and Büyükahiska, 2025) while retaining their rank-correlation identity. The resulting pooled association estimate was approximately 0.64 (95% CI [0.47, 0.77]; *I*^2^ = 87.7%; *t*-based prediction interval approximately [−0.20, 0.94]). Because Pearson r and Spearman ρ quantify different forms of association, this analysis is reported only as a sensitivity check and not as the primary correspondence estimate. Source-verified native agreement evidence was also retained separately for example, Koraishi (2024) reported ICC = 0.814 and weighted κ = 0.811, Yancey et al. (2023) reported QWK = 0.81, and Liu et al. (2025) reported QWK = 0.66. These coefficients support the presence of substantial agreement in some contexts, but they are not pooled with Pearson correlations or treated as evidence of validity by themselves.

### 5.5 Validity and construct representation findings

Construct representation was examined directly in 11 studies, but the audited quantitative evidence was too heterogeneous to support the previously reported pooled grammar- and discourse-level correlation estimates. The source-verified evidence instead shows substantial variation across constructs and metric types. For example, Mizumoto et al. (2024) reported a Spearman association of ρ = 0.79 for linguistic accuracy, whereas Yang (2024) reported Pearson correlations of *r* = 0.364 for language and *r* = 0.181 for organization. These values reflect study-specific construct differences rather than pooled domain effects. ICC/QWK-based trait results and MFRM/G-theory findings were retained on their native scales and were not converted into Pearson correlations merely to increase subgroup sample size.

Several studies reported instances in which LLMs produced scores similar to expert raters while emphasizing different textual features. Prompt sensitivity was also reported across multiple studies, with changes in prompt wording, rubric sequencing, or contextual framing associated with shifts in scoring severity and evaluative emphasis. These findings indicate variability in scoring behavior across prompting conditions, although the extent to which such variability affects construct representation remains insufficiently investigated. Most importantly, correspondence with human scores demonstrates correspondence with human scoring behavior; it does not, by itself, establish that the LLM is representing the intended construct. The removal of the unreproducible pooled grammar and discourse estimates strengthens this distinction by avoiding a false appearance of construct-level precision unsupported by the audited evidence (Section 2.2). Table 7 summarizes the construct-representation findings.

**Table 7:** Validity and construct representation findings.

Domain	Main findings
Source-verified association examples	Mizumoto et al. (2024): linguistic accuracy Spearman ρ = 0.79; Yang (2024): language *r* = 0.364 and organization *r* = 0.181. These are study-specific, not pooled domain effects.
Native agreement/measurement evidence	ICC, weighted κ/QWK, MFRM, and G-theory findings were retained on their native scales rather than converted into Pearson *r*.
Conditions reported as supporting more stable scoring	Rubric-guided prompts, constrained tasks, structured scoring criteria, and human oversight were recurrent qualitative patterns; no audited moderator meta-regression is retained for correspondence.
Construct-correspondence dissociation	Documented in multiple studies: similar human and LLM scores can coexist with different feature weighting or evaluative reasoning.
Prompt sensitivity	Small wording or framing changes can alter scoring severity and evaluative emphasis.
Primary limitation	Human-score correspondence is not independent evidence of construct representation; direct construct evidence remains sparse and heterogeneous.
Certainty level	Limited; the audited evidence does not support pooled grammar-vs.-discourse effect estimates.

### 5.6 Reliability, reproducibility, and fairness findings

#### 5.6.1 Reliability and reproducibility

Reliability-labeled evidence was reported in 18 studies, but the audit confirmed that human-score correspondence and repeated-run reliability must be treated separately. Five studies examined repeated-run reproducibility by submitting identical inputs to the same model on multiple occasions. Reported score variation ranged from negligible to differences of two or more scale points, with greater variability under open-ended prompting and for higher-order dimensions. These five studies used incompatible reproducibility metrics and were therefore synthesized narratively rather than pooled. The small source-verified direct-Pearson correspondence re-analysis (*k* = 3) cannot be characterized as demonstrating general reliability, and the native ICC/κ/QWK results likewise address agreement or reliability under specific designs rather than operational reproducibility across prompts, model versions, or deployment settings. Correspondence, agreement, repeated-run stability, prompt sensitivity, model-version stability, and operational generalizability therefore remain distinct evidential questions (Section 2.3).

Reporting transparency remained inconsistent across the corpus. Prompt documentation was incomplete in 31 studies (60%), and model-version information was absent in 14 studies (27%). Table 8 summarizes reliability and reproducibility findings.

**Table 8:** Reliability and fairness findings.

Domain	Main findings
Reported contexts with stronger score correspondence	Individual studies often reported stronger results under analytic, rubric-guided, or otherwise constrained conditions; no verified correspondence moderator meta-analysis is retained.
Reproducibility concerns	Repeated-run variability, prompt sensitivity, model-version instability, and incomplete reporting
Fairness threats examined	Measurement bias (4 studies), construct-cultural variation (2 studies)
Fairness threats unexamined	Representational bias, dialect-group comparisons, heritage speakers, script-direction effects
Common methodological limitations	Small subgroup samples, broad demographic categories, and insufficient statistical power
Non-significant findings	Cannot be interpreted as evidence of fairness, given underpowered analyses
Certainty level	Limited; current evidence insufficient to support or refute

#### 5.6.2 Fairness

Fairness was the least frequently examined inferential domain. Nine studies conducted subgroup analyses, and six provided quantitatively synthesizable fairness-related evidence. Examined subgroup variables included proficiency level (*n* = 6), L1 background (*n* = 4), discourse style (*n* = 3), and demographic category (*n* = 2). No study examined dialect-group comparisons, heritage-speaker populations, or script-direction effects.

Using the fairness taxonomy introduced in Section 2.4, four studies examined measurement bias. Two reported no statistically significant subgroup differences, whereas two reported differential scoring severity for lower-proficiency learners, particularly on discourse-level dimensions. Representational bias was not directly investigated in any included study. Construct-relevant cultural variation was examined in two studies, both of which reported qualitative evidence of differential evaluation of rhetorical patterns across cultural contexts. Subgroup analyses were generally based on modest sample sizes. The median subgroup sample size was 34 participants per group. Yamashita (2025) reported *post-hoc* power estimates below 0.40 for several non-significant fairness analyses, and Rodrigues et al. (2025) noted limited statistical power to detect effects smaller than a large magnitude in short-answer grading contexts.

Overall, the audited evidence supports cautious use of LLMs in formative and low-stakes assessment contexts, while stronger claims about operational stability, construct representation, fairness, and autonomous high-stakes deployment remain insufficiently supported. Table 9 summarizes the revised, source-verified quantitative findings, along with metric-specific and narrative evidence. For native agreement, the three strictly L2 source-verified studies include Koraishi (ICC = 0.814; weighted κ = 0.811), Yancey et al. (QWK = 0.81), and Liu, Qi, and Lu (QWK = 0.66); additional scope-caveated agreement evidence is documented in Supplementary Table 8.

**Table 9:** Audited quantitative and metric-specific findings across score correspondence, reproducibility, fairness, and washback domains.

Outcome domain	*k*	Effect metric	Pooled estimate	95% CI	Prediction interval	Heterogeneity (*I*^2^)
Source-verified direct Pearson correspondence	3	Pearson r	0.66	[0.53, 0.75]	—	70.8%
Association sensitivity	5	*r* + ρ (sensitivity)	≈0.64	[0.47, 0.77]	[−0.20, 0.94]	87.7%
Native agreement evidence	3	ICC/κ/QWK	—	—	—	—
Washback outcomes	14	Hedges' *g*	0.42	[0.27, 0.57]	[0.05, 0.78]	69.8%
Fairness subgroup comparisons	6	Narrative	—	—	—	Substantial
Repeated-run reproducibility	5	Stability indicators	—	—	—	High

### 5.7 Washback and consequential validity findings

#### 5.7.1 Quantitative Washback synthesis

Washback outcomes were synthesized across 14 studies using Hedges' *g*. The pooled effect for revision quality and short-term writing improvement under formative conditions was *g* = 0.42 (95% CI [0.27, 0.57]; *I*^2^ = 69.8%; prediction interval [0.05, 0.78]).

The review then conducted publication-bias analyses for the washback meta-analysis. Egger's regression test indicated significant funnel-plot asymmetry (intercept = 2.93, SE = 0.60, *t*_(12)_ = 4.85, *p* < 0.001), suggesting possible small-study effects. However, trim-and-fill analysis did not impute any missing studies, and the adjusted pooled effect remained substantively similar to the observed estimate (*g* = 0.39, 95% CI [0.26, 0.53]). Taken together, these findings indicate that although funnel-plot asymmetry was detected, adjustment for potentially missing studies did not materially alter the estimated washback effect or the overall interpretation of the findings.

#### 5.7.2 Qualitative washback findings

Qualitative evidence documented both positive and potentially problematic consequences of LLM-mediated feedback. Reported benefits included greater engagement with iterative revision, increased accessibility of feedback, and enhanced metalinguistic awareness when feedback was accompanied by guided reflection activities (Asadi et al., 2025; Ding, 2025; Meyer et al., 2023).

Several studies also reported cognitive offloading, acceptance of revisions without critical evaluation, and reduced metacognitive engagement during revision (Liu et al., 2024; Teng, 2024). Examples included reliance on LLM-generated revisions without explanation of the underlying changes and shorter, less reflective revision processes compared with teacher-mediated feedback conditions. No included study examined washback longitudinally beyond the duration of a specific intervention. The longest intervention lasted 16 weeks, whereas most studies reported outcomes over four to eight weeks.

**Figure 3:**
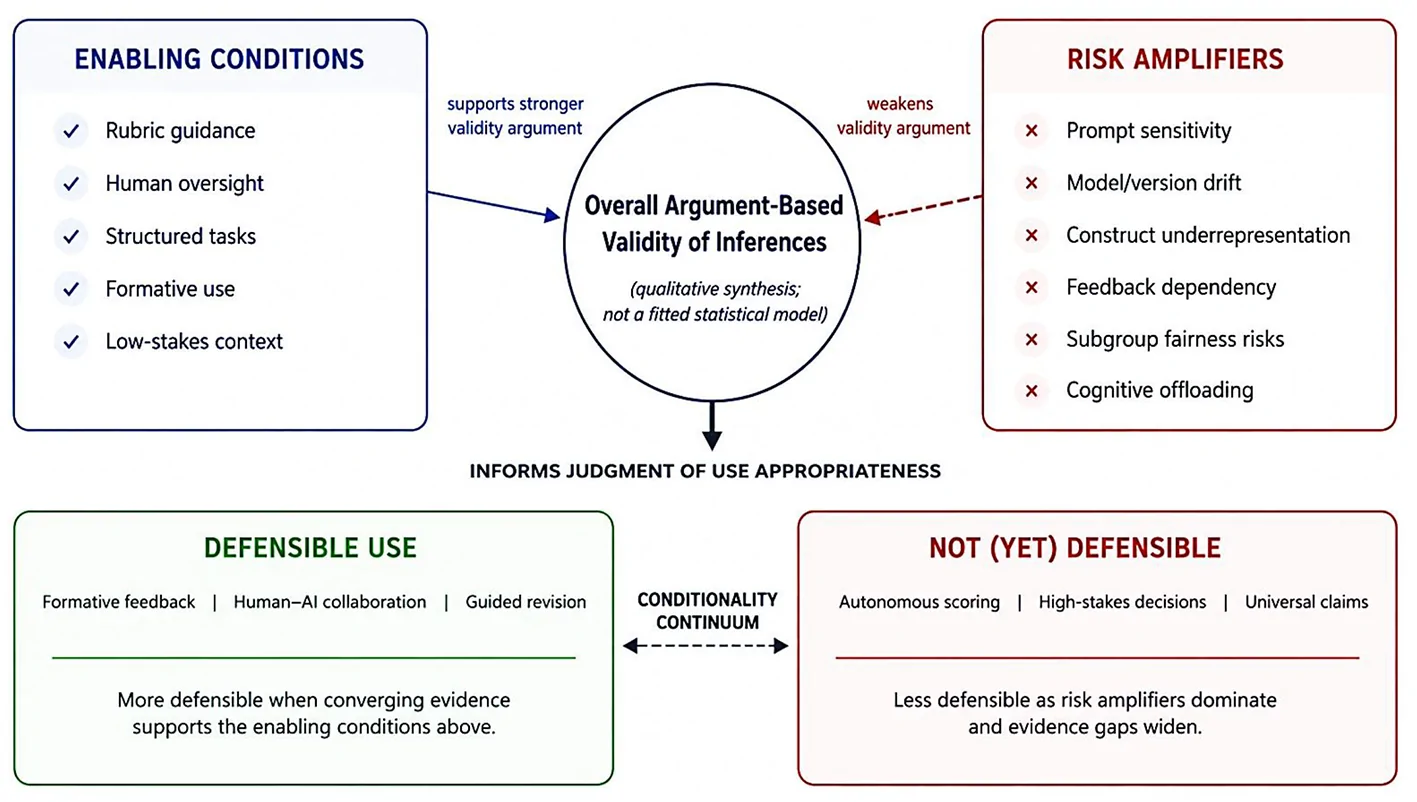
Conditional-validity framework for LLM-mediated L2 assessment.

Figure 3 qualitatively summarizes the enabling conditions presented in Sections 5.4–5.6 and the risk amplifiers discussed in Sections 6.2–6.5, and how their balance shifts the defensibility of a given use case. It is a narrative organizing diagram, not the output of a structural equation model, path analysis, or regression. The quantitative procedures retained after audit comprise the source-verified random-effects Pearson re-analysis, a rank-correlation sensitivity analysis, the washback meta-analysis, and metric-specific or narrative synthesis for outcomes that could not be defensibly harmonized. An earlier version of this figure displayed illustrative path coefficients, an *R*^2^ value, and predicted probabilities that were not produced by, or traceable to, any analysis reported in this manuscript; these were removed rather than retrofitting a methods description to unverified quantities.

Authorial agency and identity were examined qualitatively in six studies. Findings were mixed but directionally concerning: learners who used LLM feedback extensively over multiple weeks reported increased uncertainty about voice and authorship, and several described a gradual sense that the text “belonged” less to them following extensive AI-mediated revision (Teng, 2024). These findings are preliminary and methodologically limited, relying on self-report data and small samples, but they point to consequential validity concerns that warrant substantially more empirical attention, particularly given the centrality of authorship and identity to L2 writing development (Manchón, 2011). Table 10 presents the revised audited quantitative synthesis and metric-specific evidence.

**Table 10:** Summary of audited quantitative and metric-specific findings.

Outcome domain	*k*	Metric	Pooled estimate	95% CI	Prediction interval	*I* ^2^	Certainty
Source-verified direct Pearson correspondence	3	Pearson r	0.66	[0.53, 0.75]	—	70.8%	Limited
Association sensitivity	5	*r* + ρ	≈0.64	[0.47, 0.77]	[−0.20, 0.94]	87.7%	Sensitivity
Native agreement evidence	3	ICC/κ/QWK	—	—	—	—	Context-specific
Washback: revision quality	14	Hedges' *g*	0.42	[0.27, 0.57]	[0.05, 0.78]	69.8%	Moderate
Washback: teacher-mediated	8	Hedges' *g*	0.61	[0.44, 0.78]	[0.22, 0.89]	58.3%	Moderate
Washback: autonomous LLM	6	Hedges' *g*	0.28	[0.11, 0.45]	[−0.09, 0.61]	61.4%	Limited
Fairness subgroup comparisons	6	Narrative	—	—	—	Substantial	Limited
Repeated-run reproducibility	5	Stability indicators	—	—	—	High	Limited

The direct-Pearson correspondence estimate is an exploratory source-verified re-analysis based on three independent L2 studies.

## 6 Discussion

### 6.1 Overview of findings

This review synthesized evidence from 52 empirical studies through an argument-based validity framework encompassing construct representation, reliability and reproducibility, fairness, and washback. The findings support neither unconditional endorsement nor rejection of LLM-mediated assessment. Instead, they indicate that validity evidence varies systematically across assessment purposes, task characteristics, implementation conditions, and learner populations.

The source-verified direct-Pearson re-analysis yielded moderate average human–LLM score correspondence (*r* = 0.66, 95% CI [0.53, 0.75]) but substantial heterogeneity (*I*^2^ = 70.8%) across only three independent studies. Because *k* = 3, a prediction interval is not meaningfully informative. Native ICC/κ/QWK evidence showed substantial agreement in some individual studies, but these coefficients were retained on their own scales rather than collapsed into the Pearson estimate. The resulting evidence is therefore more limited than the historical composite suggested and does not support a single generalizable index of LLM scoring performance across tasks, populations, or deployment conditions.

### 6.2 Reliability without full interpretive stability

Some studies reported comparatively stable scoring under structured, rubric-aligned conditions, but the revised audit does not support treating stronger human-score correspondence as evidence of general reliability. Prompt sensitivity and repeated-run variability emerged across multiple studies, and identical inputs did not always produce identical outputs. Small changes in prompt wording, rubric sequencing, model version, or contextual framing were associated with differences in scoring behavior. Incomplete reporting of prompts and model versions compounded these patterns, limiting reproducibility across studies and operational settings.

Comparisons with human rater variability should therefore be interpreted cautiously. Although both human and AI-based assessment systems exhibit variability, the mechanisms underlying LLM inconsistency, including stochastic generation, model updates, and prompt sensitivity, differ from those typically addressed through conventional psychometric calibration procedures. The available evidence suggests greater score stability under specified conditions, but operational reliability and reproducibility remain insufficiently established.

### 6.3 Construct representation as the central validity challenge

Construct representation remains the most important unresolved validity question in LLM-mediated L2 assessment. The audited quantitative evidence is too sparse and metrically heterogeneous to support pooled grammar-vs.-discourse effect estimates. Individual studies nevertheless indicate that alignment can vary meaningfully by dimension, with some lower-inference linguistic features showing stronger correspondence than higher-order organization or discourse judgments. These study-specific patterns should not be generalized into universal construct-level performance claims.

This pattern suggests that LLM evaluations may be more sensitive to readily observable textual features in language output than to higher-order communicative constructs that depend on contextual, rhetorical, and sociocultural interpretation. Construct-correspondence dissociation findings strengthen this concern. Several studies reported cases in which LLMs produced scores similar to expert raters while emphasizing different textual characteristics, indicating that score correspondence alone cannot establish construct representation. Future validation research would benefit from approaches that directly examine the basis of LLM scoring decisions, including feature attribution analyses, convergent validity designs, and qualitative comparisons between model-generated and expert evaluative reasoning.

### 6.4 Fairness as an underdeveloped evidence domain

The fairness findings are best characterized as limited evidence rather than evidence of equitable functioning. Across the nine studies that examined subgroup comparability, sample sizes were generally small, statistical power was often limited, and several learner populations, including dialect groups, heritage speakers, and script-diverse populations, were not represented. As a result, current evidence remains insufficient to support broad claims regarding fairness across the populations likely to encounter LLM-mediated assessment in practice.

The three-category taxonomy proposed in this review- measurement bias, representational bias, and construct-relevant cultural variation- provides a framework for organizing future fairness research. Each category requires distinct forms of evidence, ranging from analyses of differential functioning and subgroup validation studies to investigations of training-data representation and cross-cultural construct comparability. Expanding fairness research across these domains remains a priority for developing defensible LLM-mediated assessment systems.

### 6.5 Washback, agency, and educational consequences

Washback findings present a more nuanced picture than either unqualified endorsement or rejection of LLM-mediated feedback. Small-to-moderate positive effects on revision quality under supervised formative conditions (*g* = 0.42) suggest meaningful instructional potential, particularly where individualized human feedback is difficult to provide at scale. The moderating effect of teacher involvement further indicates that educational benefits depend not only on the technology itself but also on the pedagogical conditions under which it is implemented. At the same time, several studies reported cognitive offloading, reduced metacognitive engagement, and increased reliance on AI-generated revisions. These findings suggest that improvements in textual performance do not necessarily correspond to equivalent gains in language development. The text improves; the learner may not. This distinction is particularly important in L2 writing, where revision is widely regarded as a central mechanism of learning and development (Manchón, 2011; Swain, 2006).

Preliminary evidence also raises concerns about authorial agency, including uncertainty about voice, ownership, and authorship during extended autonomous use. Although current evidence remains limited, these findings highlight the need to evaluate consequential validity beyond short-term revision outcomes. Longitudinal research is needed to examine how sustained exposure to LLM-mediated assessment influences learner agency, revision practices, and conceptions of writing quality over time.

### 6.6 Integrating the four domains: an argument-based, conditionally bounded view of validity

Considered together, the four inferential domains reveal a pattern not visible in any single domain alone. Human-score comparison evidence is informative but quantitatively sparse and heterogeneous once restricted to source-verifiable, metric-appropriate analyses. Construct representation evidence remains limited, particularly for higher-order communicative constructs. Fairness evidence is insufficient to draw broad inferences across diverse learner populations, and washback evidence indicates both instructional benefits and potential risks to agency and metacognitive engagement.

Read through Kane's (2013) argument-based model; these findings support a conditionally bounded validity argument rather than a universal judgment regarding LLM-mediated assessment: the same interpretive inferences that argument-based theory already specifies are better or more weakly warranted depending on the conditions of use. Validity claims are most defensible when assessment tasks are structured and rubric-aligned, prompts are transparent and standardized, human oversight remains integrated, stakes are low, and implementation conditions resemble those represented in existing validation evidence. Evidential support becomes progressively weaker as assessment moves toward higher-order communicative judgment, autonomous deployment, high-stakes decision-making, or learner populations insufficiently represented in current research.

A practical implication follows from this framework: the evidential burden increases as assessment conditions depart from those under which validity evidence has been established. Existing research provides partial support for formative, rubric-guided, and human-supervised applications. More consequential uses require substantially stronger evidence than is currently available, particularly regarding construct representation, fairness, reproducibility, and long-term educational consequences. Accordingly, this review contributes not by proposing a new form of validity, but by operationalizing established argument-based validity principles for evaluating LLM-mediated assessment across different deployment contexts.

### 6.7 Limitations and inferential boundaries

Several limitations should inform the interpretation of the present findings. The evidence base was concentrated in university-level EFL writing contexts and predominantly involved short-term interventions conducted under controlled conditions. Evidence for speaking assessment, multilingual settings beyond EFL, secondary education, heritage-language learners, integrated tasks, and long-term operational use remains limited. Accordingly, the findings are most directly applicable to the contexts represented in the current literature. In addition, incomplete reporting of prompts, model versions, parameter settings, and repeated-run procedures reduced reproducibility and comparability across studies. Although transparency coding and sensitivity analyses helped address this limitation, they cannot fully compensate for missing information in the primary studies.

Given the rapid pace of technological change, several included studies examined model versions that have since been updated or deprecated. Consequently, interpret specific performance estimates as context- and time-bound. The conditional validity framework proposed in this review is likely to be more durable than individual effect estimates, which will require ongoing re-evaluation as LLM systems evolve. Fairness also remains the least developed inferential domain. Existing evidence is insufficient to support strong conclusions regarding equitable functioning across diverse learner populations, particularly for groups that remain largely absent from current validation research.

The post-review audit also revealed an important limitation of the earlier quantitative reporting: the historical 21-study cross-metric correspondence dataset and effect-specific conversion procedure could not be independently reconstructed from the archived materials. Rather than retain an estimate that could not be transparently audited, the revised manuscript reports a smaller source-verified direct-Pearson re-analysis and separates rank correlations, ICC/κ/QWK, MFRM, and G-theory evidence by metric family. This substantially reduces the breadth of the pooled correspondence evidence and should be considered when interpreting the results. Formal publication-bias testing was therefore not conducted for correspondence, whereas the washback synthesis (*k* = 14) retained Egger's regression and trim-and-fill; its detected asymmetry should still be interpreted cautiously because of the modest number of studies. Repeated-run reproducibility (*k* = 5) was synthesized narratively because the studies used incompatible metrics and designs. Despite the comprehensive search strategy, some studies published near the search closure date may not have been captured, and restricting the review to English-language publications may have limited representation of research from other linguistic and educational contexts.

## 7 Conclusion

This systematic review and meta-analysis synthesized evidence from 52 empirical studies examining LLM-mediated L2 assessment within an argument-based validity framework (Kane, 2013). The review's principal contribution is extending argument-based validity principles to the AI-mediated assessment context and specifying the evidence base needed to sustain that argument, rather than establishing a new validity paradigm. The findings support a conditional validity framework rather than a universal interpretation of LLM assessment capability. Current evidence indicates that the defensibility of LLM-mediated assessment depends on assessment purpose, construct complexity, implementation conditions, and the extent of human oversight.

Evidence was strongest under rubric-guided, structurally constrained, formative, and human-supervised conditions, but the audited quantitative base for direct Pearson score correspondence is small. The source-verified re-analysis produced *r* = 0.66 (95% CI [0.53, 0.75]) across only three independent L2 studies, with substantial heterogeneity (*I*^2^ = 70.8%); no substantively meaningful prediction interval can be estimated at this *k*. Separate native ICC/κ/QWK findings show substantial agreement in some studies, while the washback synthesis indicates small-to-moderate improvements in revision quality under formative conditions. These findings support carefully bounded formative applications, including feedback support, first-pass scoring, and guided revision, but they do not support autonomous high-stakes scoring, universal claims of construct validity, broad fairness claims, or replacement of expert human judgment in consequential contexts. Construct representation, fairness, reproducibility, and long-term washback remain underdeveloped evidence domains, and scoring equivalence cannot be assumed across tasks, populations, or deployment conditions.

Future research should prioritize direct investigations of construct representation, adequately powered fairness studies, reproducibility research with transparent reporting and repeated-run designs, longitudinal washback studies, and validation across a broader range of assessment contexts. Advancing these areas is essential for determining not only whether LLMs can approximate human judgments, but whether those judgments support defensible educational interpretations. The principal contribution of this review is therefore not a categorical answer to whether LLMs are valid assessment tools. Rather, it is the specification of the evidential conditions under which validity claims are more or less defensible. Assessment defensibility is not a property of the technology itself, but of the validity argument supporting its use. From this perspective, the central challenge for future research is not improving correspondence alone, but strengthening the evidence required to justify assessment decisions in increasingly diverse educational contexts.

## Data Availability

The original contributions presented in the study are included in the article/Supplementary material, further inquiries can be directed to the corresponding authors.
