# Supplementary material for: Conditional validity in LLM-mediated L2 assessment: an argument-based systematic review and meta-analysis
Source: Front Res Metr Anal. 2026 Sep 11;11:1908592. doi: 10.3389/frma.2026.1908592 (PMC13612528; doi:10.3389/frma.2026.1908592)
Supplement: Supplementary file 1 [file Data_Sheet_1.docx]

**Supplementary Information**

**S1. Information Sources and Search Strategies**

Systematic searches were conducted across Web of Science Core Collection, Scopus, ERIC, PsycINFO, ProQuest Dissertations & Theses Global, Linguistics and Language Behavior Abstracts (LLBA), IEEE Xplore, ACM Digital Library, ACL Anthology, arXiv, and selected institutional repositories. These sources were chosen to ensure coverage of applied linguistics, educational assessment, and natural language processing. The temporal window and language restrictions were set according to the parameters specified in the main manuscript. Only English-language publications were retained due to the feasibility of full-text appraisal. Search strategies were structured around three concept blocks: (a) large language models and generative AI systems, (b) assessment-related constructs, and (c) second-language contexts. Platform-specific syntactic adaptations were applied to accommodate indexing conventions.

**Table S1. Search-Concept Architecture**

| Concept Block | Purpose | Representative Terms |
| --- | --- | --- |
| LLM systems | Identify generative models | ChatGPT; GPT-4; Claude; Gemini; LLaMA; Mistral |
| Assessment | Capture evaluation functions | scoring; feedback; validity; reliability; ICC; fairness |
| L2 context | Restrict to language learning | L2; EFL; ESL; multilingual; language assessment |

IEEE Xplore, ACM Digital Library, and arXiv search strings are reproduced verbatim in this appendix to permit exact replication. Equivalent logic was implemented in Web of Science, Scopus, ERIC, PsycINFO, ProQuest, and LLBA using database-specific operators.

**S2. Bibliographic Management and Deduplication**

All retrieved records were exported in RIS, BibTeX, or CSV format and imported into a reference management environment prior to screening. Deduplication followed a multi-stage algorithmic-plus-manual workflow designed to avoid both over- and under-removal. Duplicate detection was first conducted through DOI matching, followed by exact title–author–year matching. A third stage applied fuzzy string comparison to titles, with manual verification. Finally, conference-to-journal record pairs were manually examined, with the most complete report retained and linked to its associated versions.

**Table S2. Deduplication Decision Rules**

| Stage | Criterion | Rationale |
| --- | --- | --- |
| DOI match | Identical identifiers | Eliminates database overlaps |
| Exact metadata | Title + author + year | Removes indexing duplicates |
| Fuzzy matching | Near-identical titles | Captures variant spellings |
| Manual curation | Conference/journal pairs | Prevents double-counting |

Full bibliographic exports and deduplication logs are archived in the supplementary spreadsheets accompanying this article.

**S3. Screening Procedures and Exclusion Coding**

Study selection followed a two-stage process: (1) title/abstract screening and (2) full-text eligibility assessment. Two reviewers independently screened all records using a pre-piloted eligibility guide. Disagreements were resolved through discussion, with third-reviewer adjudication where required. Inter-rater agreement was computed at both screening stages. The identification, screening, eligibility, and inclusion stages are summarized in a PRISMA 2020 flow diagram in the supplementary figures. To ensure transparency, exclusion decisions were coded using standardized categories.

**Table S3. Title/Abstract Exclusion Codes**

| Code | Definition |
| --- | --- |
| TA1 | Not a second-language or language-learning context |
| TA2 | Did not involve an LLM or generative model |
| TA3 | No assessment, scoring, or feedback outcome |
| TA4 | Conceptual or commentary article |
| TA5 | Duplicate or superseded record |

**Table S4. Full-Text Exclusion Codes**

| Code | Definition |
| --- | --- |
| FT1 | Wrong AI system |
| FT2 | Not L2 assessment/feedback |
| FT3 | No empirical data |
| FT4 | Ineligible population |
| FT5 | Duplicate dataset |
| FT6 | Outside temporal scope |
| FT7 | Language not eligible |
| FT8 | Full text unobtainable |

Detailed screening logs and coded exclusion tables are supplied as spreadsheets.

**S4. Study-Level Extraction Framework**

All included studies were coded using a standardized, piloted extraction matrix covering bibliographic information, design characteristics, assessment contexts, LLM specifications, and outcome domains. The framework was designed to support both narrative synthesis and quantitative aggregation, while enabling explicit modelling of dependence among multiple effect sizes drawn from the same study.

**Table S5. Extraction Domains and Variables**

| Domain | Variables |
| --- | --- |
| Identification | author, year, outlet, DOI/URL, country |
| Design | study type, sample size, proficiency band, stakes |
| Task | modality, rubric, scoring scale |
| LLM details | model family, version, access mode, timestamp |
| Role of LLM | scoring, feedback, diagnostic, hybrid |
| Comparator | human raters, AES, teachers, alternative prompts |
| Outcomes | reliability, validity, fairness/bias, washback |
| Metrics | ICC, κ/QWK, r, G-theory, MFRM |
| Dependency | number of effects; analytic handling |

The full coded dataset is provided in the supplementary spreadsheets.

**S5. Methodological Quality Appraisal**

Methodological quality was assessed using the Mixed Methods Appraisal Tool (MMAT, 2018), allowing consistent evaluation across quantitative, qualitative, and mixed-methods designs. Two reviewers independently rated each study at the criterion level rather than computing aggregate scores, in accordance with MMAT reporting guidance. Disagreements were resolved through consensus.

**Table S6. MMAT Reporting Structure**

| Component | Description |
| --- | --- |
| Screening questions | Clear research question; appropriate data |
| Design-specific criteria | Sampling; measurement; confounding; completeness |
| Judgment scale | Yes / No / Can’t tell |
| Reporting unit | Study-by-criterion |

**S6. Quantitative Synthesis and Statistical Outputs**

Random-effects meta-analyses were conducted to account for heterogeneity across tasks, prompts, and model versions. Separate syntheses were conducted for ICC-based agreement indices, κ/QWK metrics, and correlation-based outcomes. Heterogeneity was quantified using Cochran’s Q, I², and τ², and prediction intervals were reported where appropriate. Dependence among multiple effects arising from the same study was handled using dependency-aware modeling approaches specified in the analysis scripts. Publication bias diagnostics were conducted when sufficient studies were available, including funnel-plot inspection and regression-based tests for asymmetry.

**Table S7. Statistical Output Categories Provided**

| Output Type | Description |
| --- | --- |
| Meta-analytic datasets | Effect sizes and variances |
| Model summaries | Pooled estimates and heterogeneity |
| Moderator analyses | Task type, modality, stakes |
| Diagnostics | Funnel plots; sensitivity tests |
| Reproducibility | R scripts and session logs |

All statistical artifacts are archived in the supplementary files.
