# Supplementary material for: Conditional validity in LLM-mediated L2 assessment: an argument-based systematic review and meta-analysis
Source: Front Res Metr Anal. 2026 Sep 11;11:1908592. doi: 10.3389/frma.2026.1908592 (PMC13612528; doi:10.3389/frma.2026.1908592)
Supplement: Supplementary file 2 [file Supplementary_file_1.docx]

**Appendix A**

Database-Specific Boolean Search Strings for Identifying Studies on LLM-Mediated L2 Assessment and Feedback (2022–2025)

| Database | Field Scope / Interface | Full Boolean Search String |
| --- | --- | --- |
| *Scopus* | TITLE-ABS-KEY | TITLE-ABS-KEY(( "large language model*" OR LLM* OR "generative AI" OR "generative artificial intelligence" OR ChatGPT OR "GPT-3" OR "GPT-3.5" OR "GPT-4" OR "GPT-4o" OR Claude OR Gemini OR Bard OR PaLM OR LLaMA OR Mistral OR Mixtral ) AND ( assess* OR test* OR exam* OR scor* OR rat* OR "automated scoring" OR "automated essay scoring" OR AES OR feedback OR "written corrective feedback" OR WCF OR rubric* OR valid* OR reliab* OR ICC OR kappa OR fair* OR bias OR DIF OR "measurement invariance" OR washback ) AND ( "second language" OR L2 OR "foreign language" OR EFL OR ESL OR "language learning" OR "language education" OR "L2 writing" OR "L2 speaking" )) |
| *Web of Science Core Collection* | TS (Topic) | TS=(( "large language model*" OR LLM* OR "generative AI" OR "generative artificial intelligence" OR ChatGPT OR "GPT-3" OR "GPT-3.5" OR "GPT-4" OR "GPT-4o" OR Claude OR Gemini OR Bard OR PaLM OR LLaMA OR Mistral OR Mixtral ) AND ( assess* OR test* OR exam* OR scor* OR rat* OR "automated scoring" OR "automated essay scoring" OR AES OR feedback OR "written corrective feedback" OR WCF OR rubric* OR valid* OR reliab* OR ICC OR kappa OR fair* OR bias OR DIF OR "measurement invariance" OR washback ) AND ( "second language" OR L2 OR "foreign language" OR EFL OR ESL OR "language learning" OR "L2 writing" OR "L2 speaking" )) |
| *ERIC (EBSCOhost)* | TI OR AB OR SU | (TI("large language model*" OR LLM* OR "generative AI" OR ChatGPT OR "GPT-4" OR "GPT-3.5" OR Claude OR Gemini OR Bard OR PaLM OR LLaMA OR Mistral OR Mixtral) OR AB("large language model*" OR LLM* OR "generative AI" OR ChatGPT OR "GPT-4" OR "GPT-3.5" OR Claude OR Gemini OR Bard OR PaLM OR LLaMA OR Mistral OR Mixtral)) AND (TI(assess* OR scor* OR rat* OR "automated scoring" OR feedback OR rubric* OR valid* OR reliab* OR fair* OR bias OR washback) OR AB(assess* OR scor* OR rat* OR "automated scoring" OR feedback OR rubric* OR valid* OR reliab* OR fair* OR bias OR washback) OR SU("language assessment" OR "writing assessment" OR "second language learning")) AND (TI("second language" OR L2 OR EFL OR ESL OR "L2 writing" OR "L2 speaking") OR AB("second language" OR L2 OR EFL OR ESL OR "L2 writing" OR "L2 speaking") OR SU("English as a Second Language")) |
| *APA PsycINFO (Ovid)* | .ti, ab,kw. | ("large language model*" OR LLM* OR "generative AI" OR ChatGPT OR "GPT-3" OR "GPT-3.5" OR "GPT-4" OR "GPT-4o" OR Claude OR Gemini OR Bard OR PaLM OR LLaMA OR Mistral OR Mixtral).ti, ab,kw. AND (assess* OR test* OR scor* OR rat* OR "automated scoring" OR "automated essay scoring" OR AES OR feedback OR rubric* OR valid* OR reliab* OR ICC OR kappa OR fair* OR bias OR DIF OR washback).ti, ab,kw. AND ("second language" OR L2 OR EFL OR ESL OR "language learning" OR "L2 writing" OR "L2 speaking").ti, ab,kw. |
| *LLBA (ProQuest)* | TI, AB, SU | TI, AB, SU(( "large language model*" OR LLM* OR "generative AI" OR ChatGPT OR "GPT-4" OR "GPT-3.5" OR Claude OR Gemini OR Bard OR PaLM OR LLaMA OR Mistral OR Mixtral ) AND ( assess* OR scor* OR rat* OR "automated scoring" OR "automated essay scoring" OR AES OR feedback OR rubric* OR valid* OR reliab* OR ICC OR kappa OR fair* OR bias OR DIF OR washback ) AND ( "second language" OR L2 OR EFL OR ESL OR "L2 writing" OR "L2 speaking" OR "language learning" )) |
| *ProQuest Dissertations & Theses Global* | TI, AB, SU | TI, AB, SU(( "large language model*" OR LLM* OR "generative AI" OR ChatGPT OR "GPT-4" OR "GPT-3.5" OR Claude OR Gemini OR Bard OR PaLM OR LLaMA OR Mistral OR Mixtral ) AND ( assess* OR test* OR scor* OR rat* OR "automated scoring" OR "automated essay scoring" OR AES OR feedback OR rubric* OR valid* OR reliab* OR ICC OR kappa OR fair* OR bias OR washback ) AND ( "second language" OR L2 OR EFL OR ESL OR "language learning" OR "L2 writing" OR "L2 speaking" )) |
| *IEEE Xplore* | Metadata / Abstract / Title | ("large language model*" OR LLM* OR "generative AI" OR ChatGPT OR "GPT-4" OR "GPT-3.5" OR Claude OR Gemini OR Bard OR PaLM OR LLaMA OR Mistral OR Mixtral) AND (assessment OR scoring OR "automated scoring" OR "essay scoring" OR feedback OR rubric* OR validity OR reliability OR ICC OR kappa OR fairness OR bias OR washback) AND ("second language" OR L2 OR EFL OR ESL OR "language assessment" OR "L2 writing" OR "L2 speaking") |
| *ACM Digital Library* | Advanced Search (Title/Abstract) | ("large language model*" OR LLM* OR "generative AI" OR ChatGPT OR "GPT-4" OR "GPT-3.5" OR Claude OR Gemini OR Bard OR PaLM OR LLaMA OR Mistral OR Mixtral) AND (assessment OR scoring OR "automated scoring" OR "automated essay scoring" OR feedback OR rubric* OR validity OR reliability OR ICC OR kappa OR fairness OR bias OR washback) AND ("second language" OR L2 OR EFL OR ESL OR "language assessment" OR "L2 writing" OR "L2 speaking") |
| *arXiv (API / Advanced)* | ti: and abs: | ( (ti: "large language model" OR ti: LLM OR ti: ChatGPT OR ti: "GPT-4" OR ti: "generative AI") OR (abs: "large language model" OR abs: LLM OR abs: ChatGPT OR abs: "GPT-4" OR abs: "generative AI") ) AND (abs: assessment OR abs: scoring OR abs: "automated scoring" OR abs: feedback OR abs: rubric OR abs: validity OR abs: reliability OR abs: fairness OR abs: bias OR abs: washback) AND (abs: "second language" OR abs: L2 OR abs: EFL OR abs: ESL OR abs: "language assessment" OR abs:"L2 writing" OR abs: "L2 speaking") |

**Appendix B**

PRISMA (2020). Checklist

| Section | Item No. | PRISMA 2020 Item | Addressed in This Review |
| --- | --- | --- | --- |
| TITLE | 1 | Identify the report as a systematic review | Yes – Title explicitly states *PRISMA 2020 systematic review of LLMs in L2 assessment* |
| ABSTRACT | 2 | Structured abstract | Background, objectives, data sources, eligibility, synthesis, limitations, conclusions |
| INTRODUCTION | 3 | Rationale | Yes – Justified by the rapid adoption of LLMs and unresolved concerns about validity, reliability, fairness, and washback |
|  | 4 | Objectives | Yes – To synthesize evidence on validity, reliability, fairness/bias, and washback of LLM-mediated L2 assessment |
| METHODS | 5 | Eligibility criteria | Yes – Studies (2022–2025) using LLMs for L2 assessment, AES, feedback, or scoring; journals, conferences, theses, and preprints included |
|  | 6 | Information sources | Yes – Web of Science, Scopus, ERIC, APA PsycINFO, LLBA, ProQuest Dissertations and Theses Global, IEEE Xplore, ACM Digital Library, and arXiv |
|  | 7 | Search strategy | Full Boolean strings for each database |
|  | 8 | Selection process | Yes – Deduplication → title/abstract screening → full-text or metadata screening; reasons for exclusion documented |
|  | 9 | Data collection process | Yes – Standardized extraction form; metadata/abstract-level extraction used when full text unavailable |
|  | 10a | Data items: outcomes | Yes – Reliability, validity, fairness/bias, washback |
|  | 10b | Data items: other variables | Yes – LLM type/version, task modality, comparator, prompt design, scoring framework |
|  | 11 | Risk of bias assessment | LLM-specific risks identified (prompt sensitivity, replication, model drift); formal tool reported narratively |
|  | 12 | Effect measures | Yes – ICC, κ, QWK, correlations, G-theory variance components, MFRM parameters |
|  | 13a | Synthesis methods | REML random-effects re-analysis for source-verified Pearson correspondence; Pearson+Spearman sensitivity; native ICC/κ/QWK and other psychometric metrics retained separately; washback synthesized with Hedges' g. |
|  | 13b | Methods to prepare data | Yes – Grouping by function (AES, feedback, speaking), outcome domain, and assessment context |
|  | 13c | Methods to explore heterogeneity | Yes (qualitative) – Task type, prompt design, model version, modality |
|  | 13d | Sensitivity analyses | Rank-correlation sensitivity analysis for correspondence; washback subgroup/sensitivity analyses retained where supported. |
|  | 13e | Reporting bias assessment | Formal small-study-effect testing not performed for correspondence (k=3); Egger/trim-and-fill retained for washback (k=14). |
|  | 13f | Certainty of evidence methods | Narrative certainty judgments (not formal GRADE) |
| RESULTS | 16a | Study selection | PRISMA flow diagram (n = 52) |
|  | 16b | Excluded studies | Yes – Reasons include non-assessment AI use, conceptual papers, duplicates |
|  | 17 | Study characteristics | Yes – 52 studies spanning writing, speaking, and short-answer grading |
|  | 18 | Risk of bias in studies | Yes (narrative) – Common risks summarized |
|  | 19 | Results of individual studies | Yes – Summarized narratively and in extraction tables |
|  | 20a | Summary of evidence | Yes – source-verified direct Pearson correspondence is moderate but sparse and heterogeneous; native agreement evidence is metric-specific; reproducibility evidence remains limited; validity is task- and context-dependent. |
|  | 20b | Heterogeneity | Yes – substantial heterogeneity in the direct-Pearson re-analysis and washback synthesis; prediction intervals interpreted only where meaningful. |
|  | 20c | Subgroup analyses | Demographic and dialect fairness are under-reported |
|  | 20d | Robustness of syntheses | Post-review source-level audit replaced the unreproducible historical cross-metric composite with a source-verified re-analysis and metric separation. |
| DISCUSSION | 23a | Interpretation | Yes – source-verified association evidence is limited and heterogeneous; native agreement, reproducibility, construct representation, fairness, and validity are not treated as interchangeable. |
|  | 23b | Limitations of evidence | Yes – Prompt dependence, opaque training data, small samples |
|  | 23c | Limitations of review | Yes – Reliance on metadata/abstract-level data for some studies |
|  | 23d | Implications | Yes – Guidance for assessment design, AI governance, and pedagogy |
| OTHER INFORMATION | 24 | Registration and protocol | Registration and protocol: Protocol developed a priori; not prospectively registered; materials available in Supplementary Files. |
|  | 25 | Support | Funding statement |
|  | 26 | Competing interests | AI use and author declarations |
|  | 27 | Data availability | Extraction tables and materials as supplements |

Appendix C

Protocol Deviations and Post-Review Quantitative Audit Corrections

| Protocol Component | Original Plan | Implemented Change | Rationale |
| --- | --- | --- | --- |
| Subgroup coding | Tool-type only | Added stakes level | Relevance to revised analysis |
| Effect harmonization | r only | Historical cross-metric composite removed; direct Pearson recheck (k=3), Spearman sensitivity, ICC/κ/QWK native, MFRM/G-theory separate. | Exact historical k=21 inputs and conversion rules were not reproducible; the revised approach preserves source auditability and metric distinctions. |
| Databases | 7 (Web of Science, Scopus, ERIC, APA PsycINFO, LLBA, ProQuest Dissertations and Theses Global, ACM Digital Library) | Added IEEE Xplore and arXiv | Coverage of NLP and AI-focused publication venues; final list of nine databases standardized with Section 4.4 |
